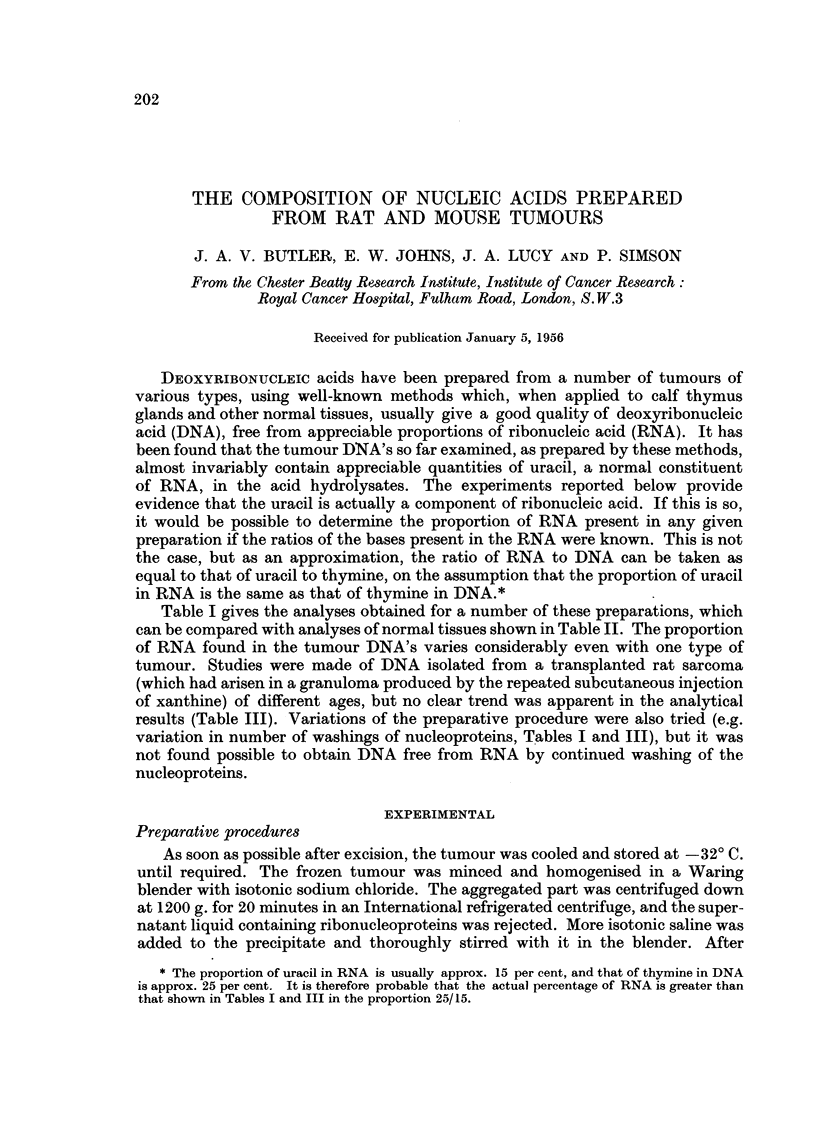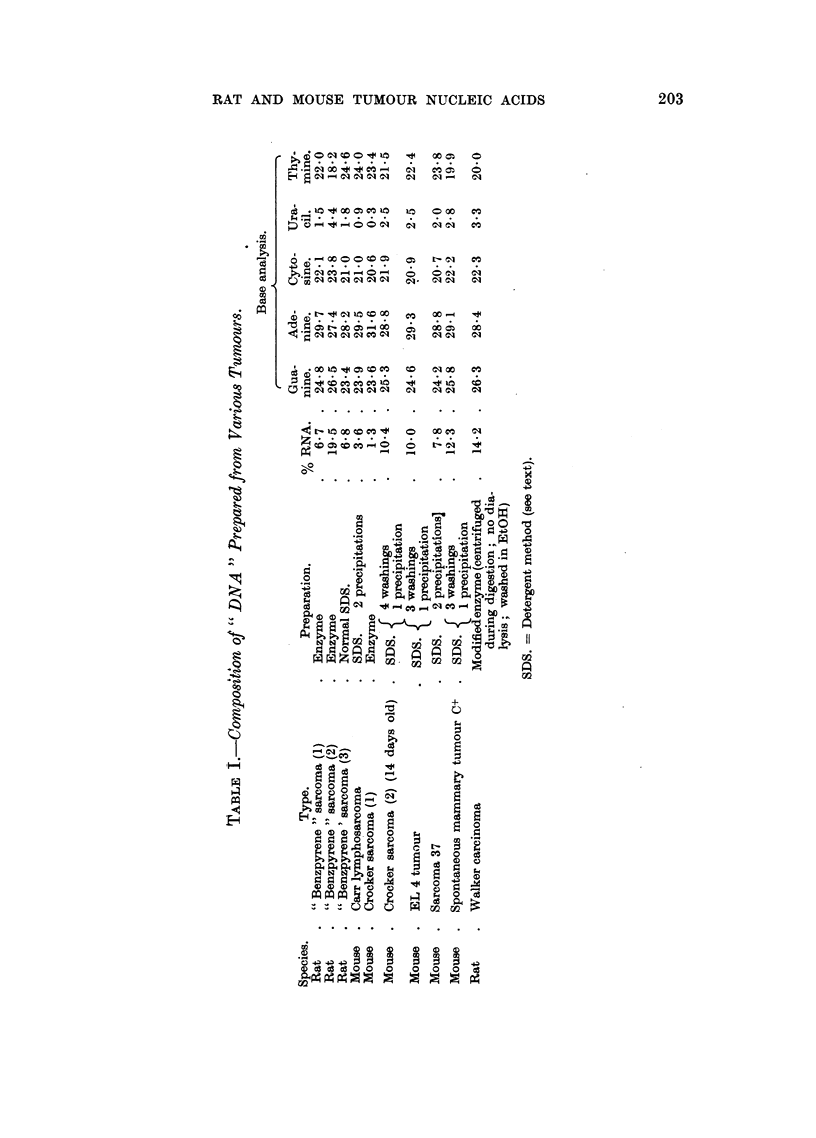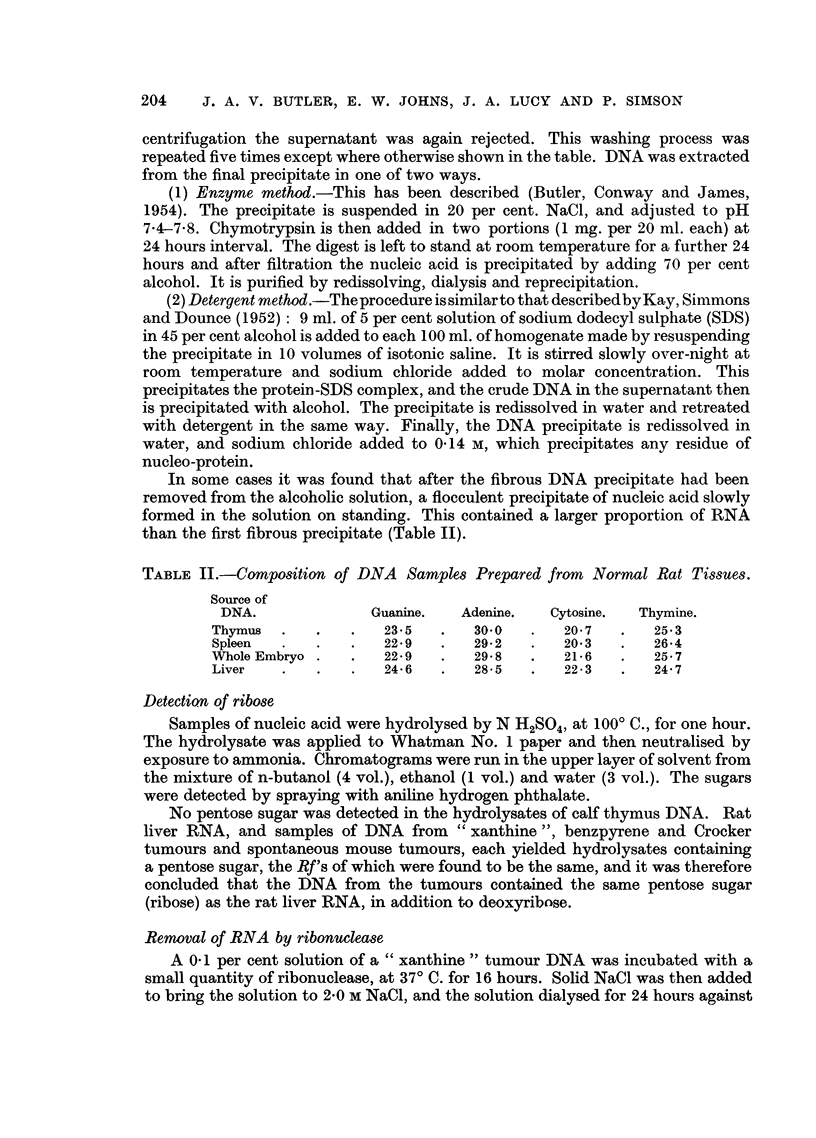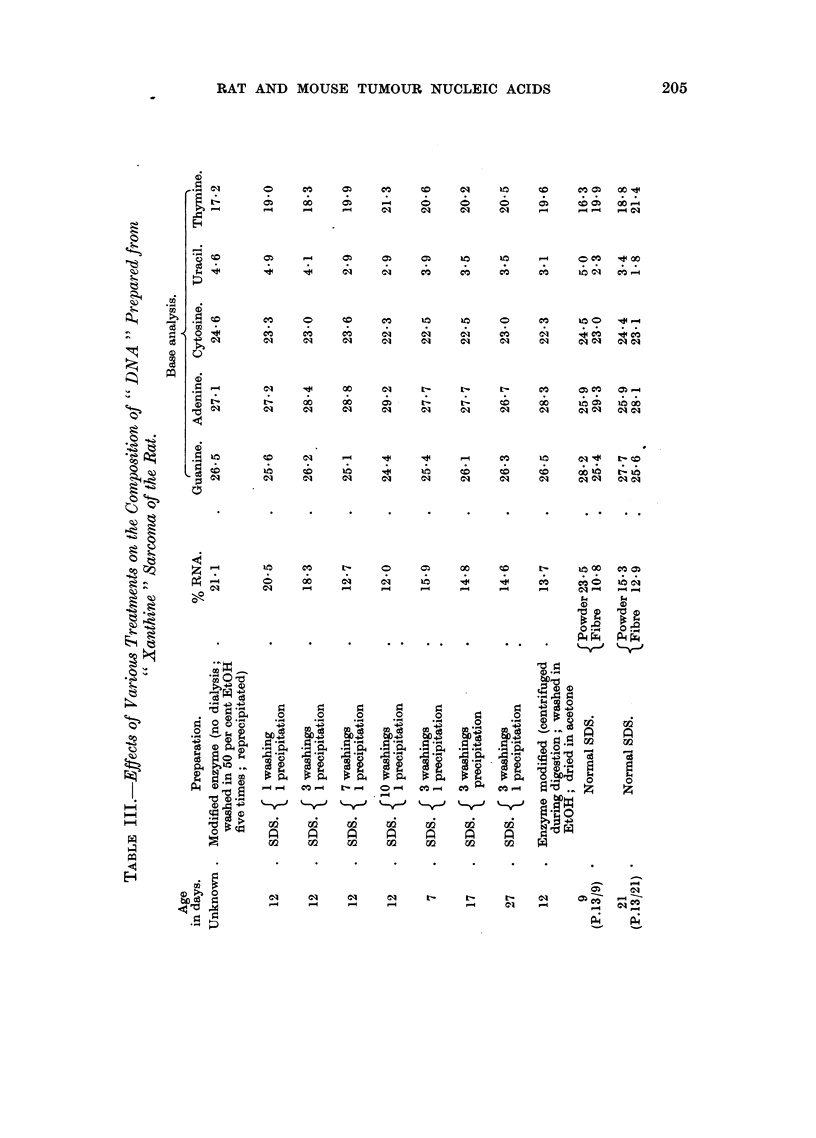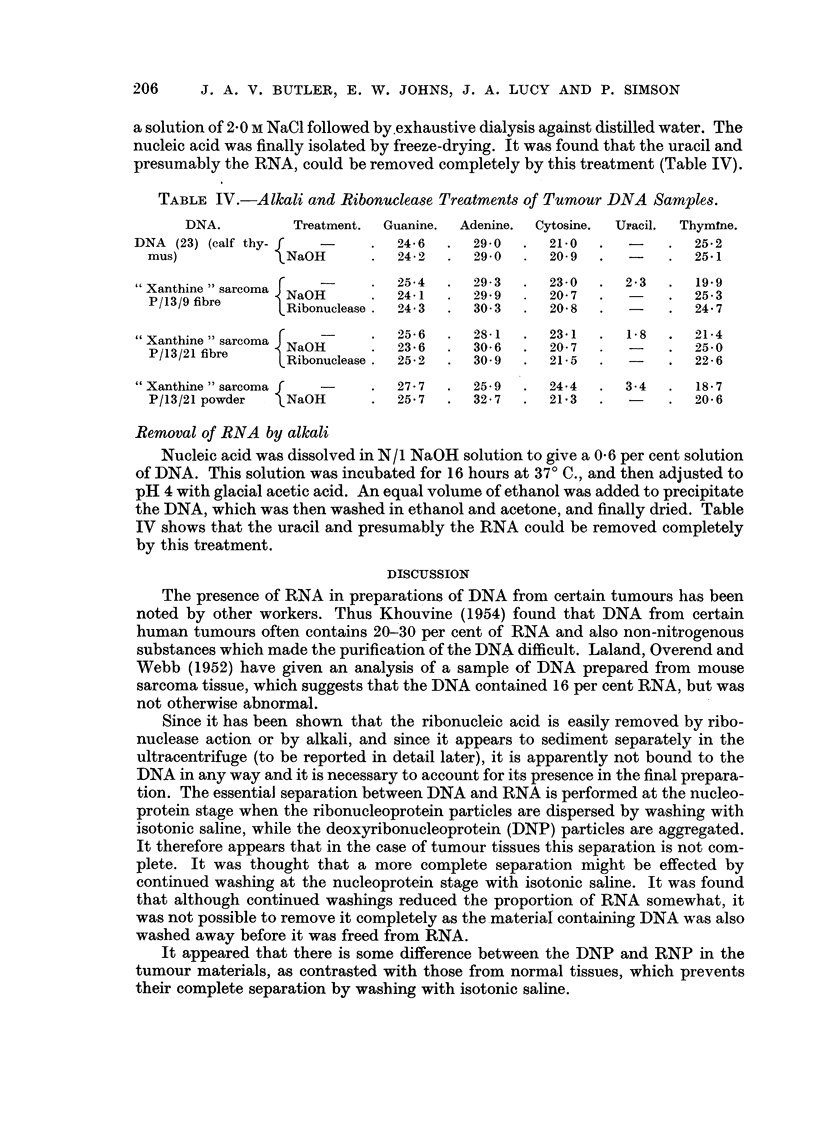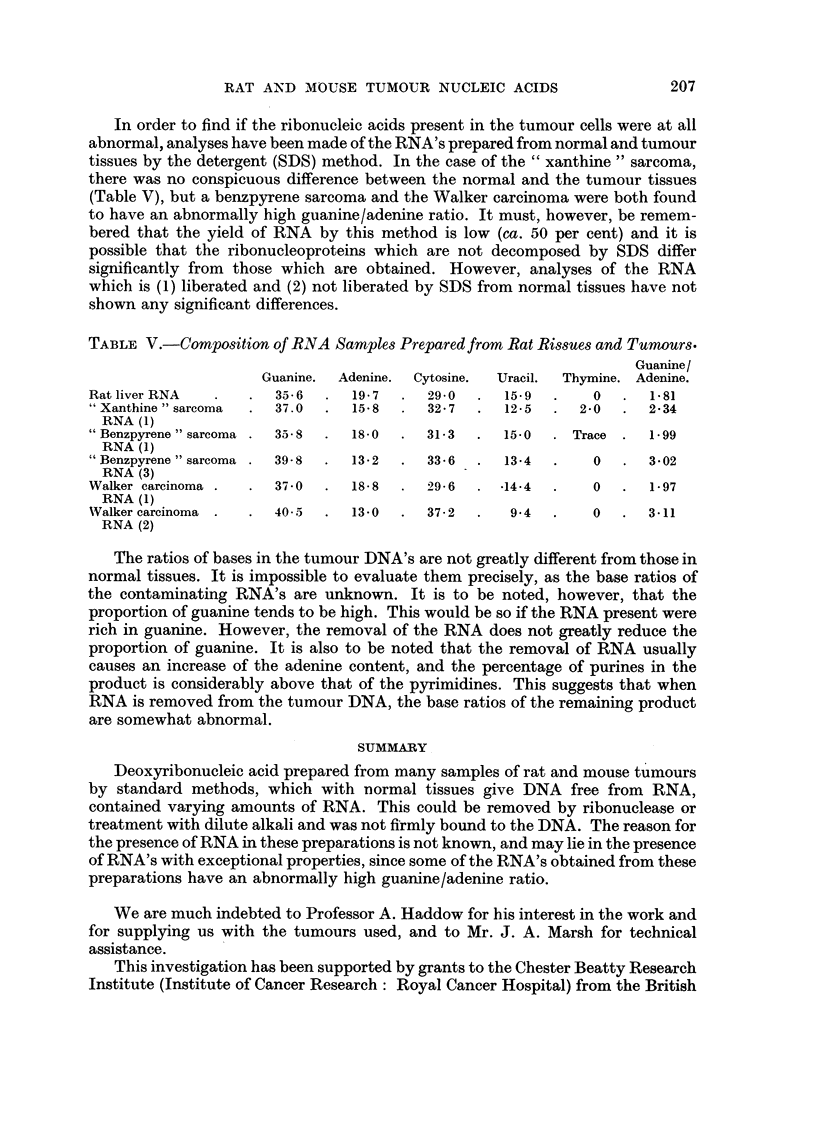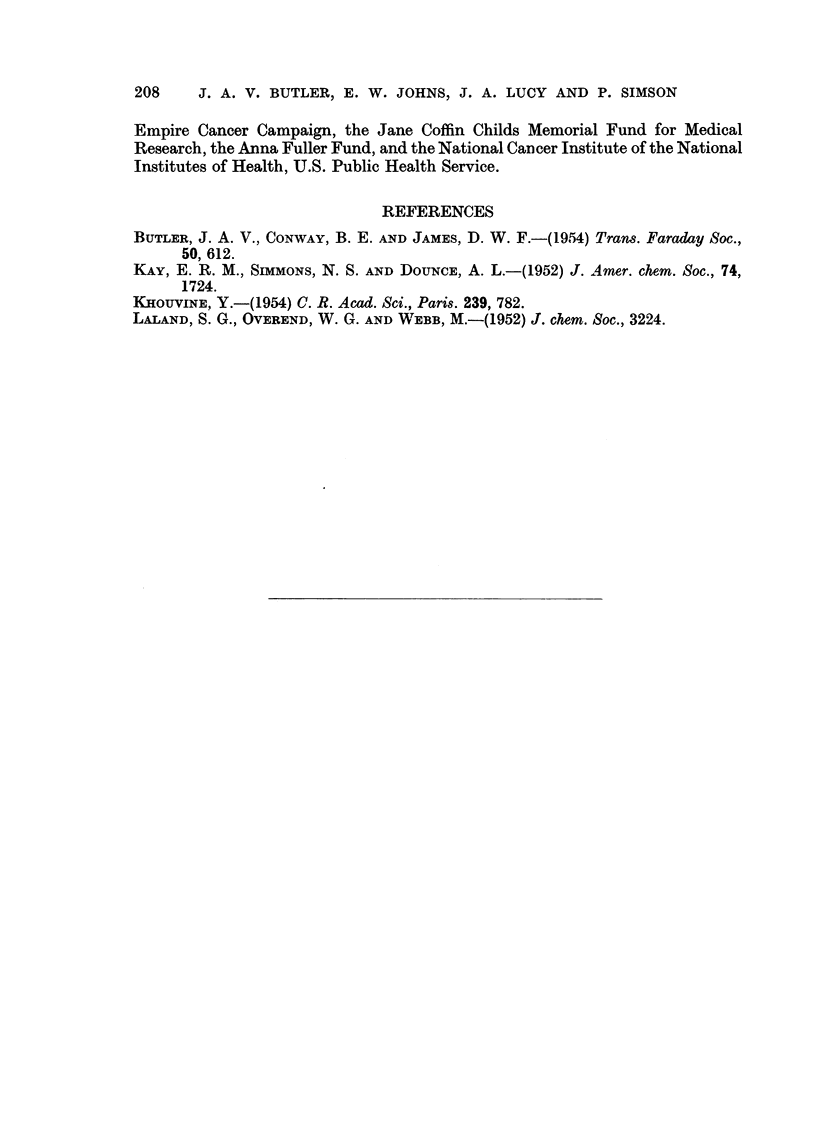# The Composition of Nucleic Acids Prepared from Rat and Mouse Tumours

**DOI:** 10.1038/bjc.1956.23

**Published:** 1956-03

**Authors:** J. A. V. Butler, E. W. Johns, J. A. Lucy, P. Simson


### THE COMPOSITION OF NUCLEIC ACIDS PREPARED FROM RAT AND MOUSE TUMOURS

J. A. V. BUTLER, E. W. JOHNS, J. A. LUCY AND P. SIMSON

*From the Chester Beatty Research Institute, Institute of Cancer Research : Royal Cancer Hospital, Fulham Road, London, S.W.3*

Received for publication January 5, 1956

DEOXYRIBONUCLEIC acids have been prepared from a number of tumours of various types, using well-known methods which, when applied to calf thymus glands and other normal tissues, usually give a good quality of deoxyribonucleic acid (DNA), free from appreciable proportions of ribonucleic acid (RNA). It has been found that the tumour DNA's so far examined, as prepared by these methods, almost invariably contain appreciable quantities of uracil, a normal constituent of RNA, in the acid hydrolysates. The experiments reported below provide evidence that the uracil is actually a component of ribonucleic acid. If this is so, it would be possible to determine the proportion of RNA present in any given preparation if the ratios of the bases present in the RNA were known. This is not the case, but as an approximation, the ratio of RNA to DNA can be taken as equal to that of uracil to thymine, on the assumption that the proportion of uracil in RNA is the same as that of thymine in DNA.*

Table I gives the analyses obtained for a number of these preparations, which can be compared with analyses of normal tissues shown in Table II. The proportion of RNA found in the tumour DNA's varies considerably even with one type of tumour. Studies were made of DNA isolated from a transplanted rat sarcoma (which had arisen in a granuloma produced by the repeated subcutaneous injection of xanthine) of different ages, but no clear trend was apparent in the analytical results (Table III). Variations of the preparative procedure were also tried (e.g. variation in number of washings of nucleoproteins, Tables I and III), but it was not found possible to obtain DNA free from RNA by continued washing of the nucleoproteins.

### EXPERIMENTAL

### *Preparative procedures*

As soon as possible after excision, the tumour was cooled and stored at —32° C. until required. The frozen tumour was minced and homogenised in a Waring blender with isotonic sodium chloride. The aggregated part was centrifuged down at 1200 g. for 20 minutes in an International refrigerated centrifuge, and the supernatant liquid containing ribonucleoproteins was rejected. More isotonic saline was added to the precipitate and thoroughly stirred with it in the blender. After

* The proportion of uracil in RNA is usually approx. 15 per cent, and that of thymine in DNA is approx. 25 per cent. It is therefore probable that the actual percentage of RNA is greater than that shown in Tables I and III in the proportion 25/15.

TABLE I.—*Composition of " DNA " Prepared from Various Tumours.*

| Species. | Type. | Preparation. | % RNA. | Base analysis. Guanine. | Adenine. | Cytosine. | Uracil. | Thymine. |
|---|---|---|---|---|---|---|---|---|
| Rat | " Benzpyrene " sarcoma (1) | Enzyme | 6·7 | 24·8 | 29·7 | 22·1 | 1·5 | 22·0 |
| Rat | " Benzpyrene " sarcoma (2) | Enzyme | 19·5 | 26·5 | 27·4 | 23·8 | 4·4 | 18·2 |
| Rat | " Benzpyrene ' sarcoma (3) | Normal SDS. | 6·8 | 23·4 | 28·2 | 21·0 | 1·8 | 24·6 |
| Mouse | Carr lymphosarcoma | SDS. 2 precipitations | 3·6 | 23·9 | 29·5 | 21·0 | 0·9 | 24·0 |
| Mouse | Crocker sarcoma (1) | Enzyme | 1·3 | 23·6 | 31·6 | 20·6 | 0·3 | 23·4 |
| Mouse | Crocker sarcoma (2) (14 days old) | SDS. { 4 washings, 1 precipitation | 10·4 | 25·3 | 28·8 | 21·9 | 2·5 | 21·5 |
| Mouse | EL 4 tumour | SDS. { 3 washings, 1 precipitation | 10·0 | 24·6 | 29·3 | 20·9 | 2·5 | 22·4 |
| Mouse | Sarcoma 37 | SDS. 2 precipitations] | 7·8 | 24·2 | 28·8 | 20·7 | 2·0 | 23·8 |
| Mouse | Spontaneous mammary tumour C+ | SDS. { 3 washings, 1 precipitation | 12·3 | 25·8 | 29·1 | 22·2 | 2·8 | 19·9 |
| Rat | Walker carcinoma | Modified enzyme (centrifuged during digestion ; no dialysis ; washed in EtOH) | 14·2 | 26·3 | 28·4 | 22·3 | 3·3 | 20·0 |

SDS. = Detergent method (see text).

centrifugation the supernatant was again rejected. This washing process was repeated five times except where otherwise shown in the table. DNA was extracted from the final precipitate in one of two ways.

(1) *Enzyme method.*—This has been described (Butler, Conway and James, 1954). The precipitate is suspended in 20 per cent. NaCl, and adjusted to pH 7·4–7·8. Chymotrypsin is then added in two portions (1 mg. per 20 ml. each) at 24 hours interval. The digest is left to stand at room temperature for a further 24 hours and after filtration the nucleic acid is precipitated by adding 70 per cent alcohol. It is purified by redissolving, dialysis and reprecipitation.

(2) *Detergent method.*—The procedure is similar to that described by Kay, Simmons and Dounce (1952) : 9 ml. of 5 per cent solution of sodium dodecyl sulphate (SDS) in 45 per cent alcohol is added to each 100 ml. of homogenate made by resuspending the precipitate in 10 volumes of isotonic saline. It is stirred slowly over-night at room temperature and sodium chloride added to molar concentration. This precipitates the protein-SDS complex, and the crude DNA in the supernatant then is precipitated with alcohol. The precipitate is redissolved in water and retreated with detergent in the same way. Finally, the DNA precipitate is redissolved in water, and sodium chloride added to 0·14 M, which precipitates any residue of nucleo-protein.

In some cases it was found that after the fibrous DNA precipitate had been removed from the alcoholic solution, a flocculent precipitate of nucleic acid slowly formed in the solution on standing. This contained a larger proportion of RNA than the first fibrous precipitate (Table II).

TABLE II.—*Composition of DNA Samples Prepared from Normal Rat Tissues.*

| Source of DNA. | Guanine. | Adenine. | Cytosine. | Thymine. |
|---|---|---|---|---|
| Thymus | 23·5 | 30·0 | 20·7 | 25·3 |
| Spleen | 22·9 | 29·2 | 20·3 | 26·4 |
| Whole Embryo | 22·9 | 29·8 | 21·6 | 25·7 |
| Liver | 24·6 | 28·5 | 22·3 | 24·7 |

*Detection of ribose*

Samples of nucleic acid were hydrolysed by N $H_2SO_4$, at 100° C., for one hour. The hydrolysate was applied to Whatman No. 1 paper and then neutralised by exposure to ammonia. Chromatograms were run in the upper layer of solvent from the mixture of n-butanol (4 vol.), ethanol (1 vol.) and water (3 vol.). The sugars were detected by spraying with aniline hydrogen phthalate.

No pentose sugar was detected in the hydrolysates of calf thymus DNA. Rat liver RNA, and samples of DNA from " xanthine ", benzpyrene and Crocker tumours and spontaneous mouse tumours, each yielded hydrolysates containing a pentose sugar, the *Rf*'s of which were found to be the same, and it was therefore concluded that the DNA from the tumours contained the same pentose sugar (ribose) as the rat liver RNA, in addition to deoxyribose.

*Removal of RNA by ribonuclease*

A 0·1 per cent solution of a " xanthine " tumour DNA was incubated with a small quantity of ribonuclease, at 37° C. for 16 hours. Solid NaCl was then added to bring the solution to 2·0 M NaCl, and the solution dialysed for 24 hours against

TABLE III.—*Effects of Various Treatments on the Composition of " DNA " Prepared from " Xanthine " Sarcoma of the Rat.*

| Age in days. | Preparation. | % RNA. | Base analysis. Guanine. | Adenine. | Cytosine. | Uracil. | Thymine. |
|---|---|---|---|---|---|---|---|
| Unknown | Modified enzyme (no dialysis; washed in 50 per cent EtOH five times; reprecipitated) | 21·1 | 26·5 | 27·1 | 24·6 | 4·6 | 17·2 |
| 12 | SDS. { 1 washing, 1 precipitation | 20·5 | 25·6 | 27·2 | 23·3 | 4·9 | 19·0 |
| 12 | SDS. { 3 washings, 1 precipitation | 18·3 | 26·2 | 28·4 | 23·0 | 4·1 | 18·3 |
| 12 | SDS. { 7 washings, 1 precipitation | 12·7 | 25·1 | 28·8 | 23·6 | 2·9 | 19·9 |
| 12 | SDS. { 10 washings, 1 precipitation | 12·0 | 24·4 | 29·2 | 22·3 | 2·9 | 21·3 |
| 7 | SDS. { 3 washings, 1 precipitation | 15·9 | 25·4 | 27·7 | 22·5 | 3·9 | 20·6 |
| 17 | SDS. { 3 washings, precipitation | 14·8 | 26·1 | 27·7 | 22·5 | 3·5 | 20·2 |
| 27 | SDS. { 3 washings, 1 precipitation | 14·6 | 26·3 | 26·7 | 23·0 | 3·5 | 20·5 |
| 12 | Enzyme modified (centrifuged during digestion; washed in EtOH; dried in acetone | 13·7 | 26·5 | 28·3 | 22·3 | 3·1 | 19·6 |
| 9 (P.13/9) | Normal SDS. | Powder 23·5 | 28·2 | 25·9 | 24·5 | 5·0 | 16·3 |
| | | Fibre 10·8 | 25·4 | 29·3 | 23·0 | 2·3 | 19·9 |
| 21 (P.13/21) | Normal SDS. | Powder 15·3 | 27·7 | 25·9 | 24·4 | 3·4 | 18·8 |
| | | Fibre 12·9 | 25·6 | 28·1 | 23·1 | 1·8 | 21·4 |

a solution of 2·0 M NaCl followed by exhaustive dialysis against distilled water. The nucleic acid was finally isolated by freeze-drying. It was found that the uracil and presumably the RNA, could be removed completely by this treatment (Table IV).

TABLE IV.—*Alkali and Ribonuclease Treatments of Tumour DNA Samples.*

| DNA. | Treatment. | Guanine. | Adenine. | Cytosine. | Uracil. | Thymine. |
|---|---|---|---|---|---|---|
| DNA (23) (calf thymus) | — | 24·6 | 29·0 | 21·0 | — | 25·2 |
| | NaOH | 24·2 | 29·0 | 20·9 | — | 25·1 |
| " Xanthine " sarcoma P/13/9 fibre | — | 25·4 | 29·3 | 23·0 | 2·3 | 19·9 |
| | NaOH | 24·1 | 29·9 | 20·7 | — | 25·3 |
| | Ribonuclease | 24·3 | 30·3 | 20·8 | — | 24·7 |
| " Xanthine " sarcoma P/13/21 fibre | — | 25·6 | 28·1 | 23·1 | 1·8 | 21·4 |
| | NaOH | 23·6 | 30·6 | 20·7 | — | 25·0 |
| | Ribonuclease | 25·2 | 30·9 | 21·5 | — | 22·6 |
| " Xanthine " sarcoma P/13/21 powder | — | 27·7 | 25·9 | 24·4 | 3·4 | 18·7 |
| | NaOH | 25·7 | 32·7 | 21·3 | — | 20·6 |

*Removal of RNA by alkali*

Nucleic acid was dissolved in N/1 NaOH solution to give a 0·6 per cent solution of DNA. This solution was incubated for 16 hours at 37° C., and then adjusted to pH 4 with glacial acetic acid. An equal volume of ethanol was added to precipitate the DNA, which was then washed in ethanol and acetone, and finally dried. Table IV shows that the uracil and presumably the RNA could be removed completely by this treatment.

### DISCUSSION

The presence of RNA in preparations of DNA from certain tumours has been noted by other workers. Thus Khouvine (1954) found that DNA from certain human tumours often contains 20–30 per cent of RNA and also non-nitrogenous substances which made the purification of the DNA difficult. Laland, Overend and Webb (1952) have given an analysis of a sample of DNA prepared from mouse sarcoma tissue, which suggests that the DNA contained 16 per cent RNA, but was not otherwise abnormal.

Since it has been shown that the ribonucleic acid is easily removed by ribonuclease action or by alkali, and since it appears to sediment separately in the ultracentrifuge (to be reported in detail later), it is apparently not bound to the DNA in any way and it is necessary to account for its presence in the final preparation. The essential separation between DNA and RNA is performed at the nucleoprotein stage when the ribonucleoprotein particles are dispersed by washing with isotonic saline, while the deoxyribonucleoprotein (DNP) particles are aggregated. It therefore appears that in the case of tumour tissues this separation is not complete. It was thought that a more complete separation might be effected by continued washing at the nucleoprotein stage with isotonic saline. It was found that although continued washings reduced the proportion of RNA somewhat, it was not possible to remove it completely as the material containing DNA was also washed away before it was freed from RNA.

It appeared that there is some difference between the DNP and RNP in the tumour materials, as contrasted with those from normal tissues, which prevents their complete separation by washing with isotonic saline.

In order to find if the ribonucleic acids present in the tumour cells were at all abnormal, analyses have been made of the RNA's prepared from normal and tumour tissues by the detergent (SDS) method. In the case of the " xanthine " sarcoma, there was no conspicuous difference between the normal and the tumour tissues (Table V), but a benzpyrene sarcoma and the Walker carcinoma were both found to have an abnormally high guanine/adenine ratio. It must, however, be remembered that the yield of RNA by this method is low (*ca.* 50 per cent) and it is possible that the ribonucleoproteins which are not decomposed by SDS differ significantly from those which are obtained. However, analyses of the RNA which is (1) liberated and (2) not liberated by SDS from normal tissues have not shown any significant differences.

TABLE V.—*Composition of RNA Samples Prepared from Rat Rissues and Tumours.*

| | Guanine. | Adenine. | Cytosine. | Uracil. | Thymine. | Guanine/ Adenine. |
|---|---|---|---|---|---|---|
| Rat liver RNA | 35·6 | 19·7 | 29·0 | 15·9 | 0 | 1·81 |
| " Xanthine " sarcoma RNA (1) | 37.0 | 15·8 | 32·7 | 12·5 | 2·0 | 2·34 |
| " Benzpyrene " sarcoma RNA (1) | 35·8 | 18·0 | 31·3 | 15·0 | Trace | 1·99 |
| " Benzpyrene " sarcoma RNA (3) | 39·8 | 13·2 | 33·6 | 13·4 | 0 | 3·02 |
| Walker carcinoma RNA (1) | 37·0 | 18·8 | 29·6 | ·14·4 | 0 | 1·97 |
| Walker carcinoma RNA (2) | 40·5 | 13·0 | 37·2 | 9·4 | 0 | 3·11 |

The ratios of bases in the tumour DNA's are not greatly different from those in normal tissues. It is impossible to evaluate them precisely, as the base ratios of the contaminating RNA's are unknown. It is to be noted, however, that the proportion of guanine tends to be high. This would be so if the RNA present were rich in guanine. However, the removal of the RNA does not greatly reduce the proportion of guanine. It is also to be noted that the removal of RNA usually causes an increase of the adenine content, and the percentage of purines in the product is considerably above that of the pyrimidines. This suggests that when RNA is removed from the tumour DNA, the base ratios of the remaining product are somewhat abnormal.

### SUMMARY

Deoxyribonucleic acid prepared from many samples of rat and mouse tumours by standard methods, which with normal tissues give DNA free from RNA, contained varying amounts of RNA. This could be removed by ribonuclease or treatment with dilute alkali and was not firmly bound to the DNA. The reason for the presence of RNA in these preparations is not known, and may lie in the presence of RNA's with exceptional properties, since some of the RNA's obtained from these preparations have an abnormally high guanine/adenine ratio.

We are much indebted to Professor A. Haddow for his interest in the work and for supplying us with the tumours used, and to Mr. J. A. Marsh for technical assistance.

This investigation has been supported by grants to the Chester Beatty Research Institute (Institute of Cancer Research : Royal Cancer Hospital) from the British

Empire Cancer Campaign, the Jane Coffin Childs Memorial Fund for Medical Research, the Anna Fuller Fund, and the National Cancer Institute of the National Institutes of Health, U.S. Public Health Service.

### REFERENCES

BUTLER, J. A. V., CONWAY, B. E. AND JAMES, D. W. F.—(1954) *Trans. Faraday Soc.*, **50**, 612.
KAY, E. R. M., SIMMONS, N. S. AND DOUNCE, A. L.—(1952) *J. Amer. chem. Soc.*, **74**, 1724.
KHOUVINE, Y.—(1954) *C. R. Acad. Sci., Paris.* **239**, 782.
LALAND, S. G., OVEREND, W. G. AND WEBB, M.—(1952) *J. chem. Soc.*, 3224.